# Dual‐Target Repurposing Against Chikungunya Virus: Molecular Docking and Dynamics Reveal Bictegravir and Oxacillin as Promising Leads

**DOI:** 10.1155/bmri/1672620

**Published:** 2026-11-04

**Authors:** Asef Raj, Humair Bin Md Omer, Mim Deb Roy, Aisha Siddika, Sabrina Sharmin

**Affiliations:** ^1^ School of Pharmacy BRAC University Dhaka Bangladesh bracu.ac.bd; ^2^ Department of Chemistry Binghamton University The State University of New York Binghamton New York USA suny.edu; ^3^ Department of Pharmaceutical Chemistry University of Technology Mara Puncak Alam Malaysia uitm.edu.my

**Keywords:** capsid, chikungunya, dynamics, nsP2, repurposing

## Abstract

Chikungunya virus (CHIKV) remains an important re‐emerging arboviral threat, yet no specific antiviral therapy is currently available. This study used a structure‐based drug‐repurposing approach to identify FDA and internationally approved drugs with potential activity against two therapeutically relevant CHIKV targets: nsP2 protease (essential for nonstructural polyprotein processing) and capsid protein (essential for viral nucleocapsid formation). Molecular docking was performed against both proteins, followed by 100‐ns molecular dynamics (MD) simulations of the two highest ranked compounds for each target to assess complex stability. Against nsP2 protease, bictegravir showed the most favorable docking score (−9.5 kcal/mol), followed by simeprevir (−9.3 kcal/mol) and glecaprevir (−9.2 kcal/mol). Against the capsid protein, trovafloxacin ranked first (−8.8 kcal/mol), followed by oxacillin (−8.7 kcal/mol) and paritaprevir (−8.6 kcal/mol). For context, the reference cysteine protease inhibitor E‐64 and the pocket‐validated ligand ethyl 3‐aminobenzoate (EAB) were docked under the identical protocol, and they scored −6.1 and −6.0 kcal/mol against nsP2 protease and capsid protein, respectively, both weaker than every top‐ranked hit identified in this study. MD simulation showed that the bictegravir–nsP2 complex maintained persistently negative total interaction energy, generally within −50 to −30 kcal/mol, with Rg fluctuating from approximately 20.6 to 21.8 Å and RMSD stabilizing initially at 1.2–1.8 Å before reaching a later plateau of 3.0–3.8 Å. By contrast, the simeprevir–nsP2 complex displayed a less uniform energetic profile. For the capsid target, the oxacillin complex remained comparatively restrained, with Rg largely within 22.4–23.2 Å and RMSD mostly within 1.4–2.0 Å, whereas the trovafloxacin complex showed greater conformational drift, with RMSD rising to nearly 2.8–3.1 Å. These computational findings prioritize bictegravir and oxacillin for further investigation against CHIKV nsP2 protease and capsid protein, respectively. No biological validation was performed; target inhibition and antiviral activity therefore remain unconfirmed and require biochemical and cell‐based evaluation.

## 1. Introduction

Chikungunya virus (CHIKV), a mosquito‐borne arthritogenic alphavirus, has re‐emerged as a pressing public health concern driven by its rapid geographic spread, epidemic potential, and the debilitating musculoskeletal disease it triggers. As of December 2024, the World Health Organization documented local transmission across 119 countries and territories. This highlights a critical shift that CHIKV is no longer a regionally confined pathogen, but rather a firmly established global threat [1]. CHIKV infection typically presents with abrupt fever, rash, and severe polyarthralgia, and although many patients recover from the acute febrile phase, joint pain may persist for weeks, months, or even years in a substantial proportion of cases [2]. This extended clinical burden compounded by occasional neurological, cardiac, and ocular complications in vulnerable populations demonstrates that chikungunya is far more than a brief, self‐resolving viral illness [3].

Despite this burden, treatment remains largely supportive. Current management focuses on hydration, analgesics, and antipyretics, whereas no specific antiviral drug has yet been approved for CHIKV infection [4]. This therapeutic gap has encouraged renewed interest in antiviral repurposing, particularly the screening of approved drugs whose pharmacokinetic properties, toxicology profiles, and clinical safety are already partly defined. CHIKV‐specific repurposing studies have already shown that this is not merely a theoretical shortcut. Several approved drugs have demonstrated measurable anti‐CHIKV activity in experimental systems. A study on 14 FDA‐approved drugs further highlighted the practical value of combining in vitro and in silico prioritization for candidate selection [5]. Among clinically used antivirals, sofosbuvir is especially notable because it inhibited CHIKV replication in cell culture and showed protective activity in mouse models, including reduction of CHIKV‐induced paw edema [6]. These findings support the broader idea that approved antiviral scaffolds can retain useful activity outside their original viral indication and may therefore offer a faster translational route than de novo discovery [7, 8].

CHIKV is a positive‐sense single‐stranded RNA virus of approximately 11.9 kb that encodes four nonstructural proteins (nsP1–nsP4) and the structural proteins capsid, E3, E2, 6K, and E1 [9]. Because these proteins drive distinct but tightly connected stages of the viral life cycle, target selection is central to rational antiviral design. In this context, nsP2 and capsid are particularly attractive because they represent two mechanistically different vulnerabilities—one sits at the heart of viral RNA replication and host shutoff, whereas the other is essential for genome packaging, nucleocapsid organization, and virion assembly [4, 10]. Screening compounds against both proteins therefore offers a biologically meaningful way to identify candidates with broader functional relevance than single‐target hits. This is especially valuable in CHIKV, where the most promising antiviral strategy may be one that interferes with more than one critical step of the viral cycle [7, 11].

Among CHIKV proteins, nsP2 has emerged as one of the most compelling antiviral targets. It is the largest nonstructural protein and performs multiple indispensable roles, including RNA helicase and RNA triphosphatase functions within its N‐terminal region and cysteine protease activity within its C‐terminal domain. These enzymatic activities are required for polyprotein processing, viral RNA replication, and transcription [4, 12]. Beyond replication, nsP2 also acts as a virulence factor. Alphavirus nsP2 proteins, including that of CHIKV, have been linked to host transcriptional and translational shutoff and suppression of interferon‐mediated antiviral responses, thereby helping the virus evade innate immunity [13–15]. The relevance of nsP2 as a drug target is reinforced by recent inhibitor studies. A 2024 report identified a cell‐active inhibitor of CHIKV nsP2 protease, and another study showed that withaferin A inhibits nsP2 protease activity and reduces viral infection in cell culture and mouse models [16, 17]. These findings argue that nsP2 is not only biologically central but also chemically tractable.

The structural capsid protein provides a second, complementary target. Capsid is classically recognized for packaging the viral genome and forming the nucleocapsid core, but evidence shows that its contribution to CHIKV biology is broader than simple structural support. The N‐terminal region interacts with viral RNA, whereas the C‐terminal protease domain contains a conserved hydrophobic pocket that binds the cytoplasmic tail of E2, an interaction that is essential for virion assembly [18, 19]. This capsid domain adopts a chymotrypsin‐like protease fold and contains a druggable hydrophobic pocket that can accommodate small molecules [20]. This is important because capsid‐directed inhibition could disrupt core assembly or E2‐capsid coupling, thereby impairing formation of infectious particles. Picolinic acid was shown to bind the conserved hydrophobic pocket of CHIKV capsid and inhibit viral replication, providing direct proof that capsid is pharmacologically actionable [21]. Capsid also undergoes active nuclear‐cytoplasmic trafficking, containing both nuclear localization and export signals, and mutation of its nucleolar localization sequence attenuated replication in mammalian and mosquito cells and markedly reduced disease in mice [22, 23]. In parallel, capsid–viral RNA interactions have been shown to regulate translation‐related events during replication, again pointing to functions that extend beyond virion architecture alone [10].

Given this biology, a dual‐target in silico workflow centered on nsP2 and capsid is well justified. Molecular docking offers an efficient first‐pass strategy to prioritize approved antivirals according to their ability to occupy catalytically or structurally relevant pockets, whereas molecular dynamics (MD) simulation adds a second layer of confidence by testing whether the docked complex remains stable over time under near‐physiological conditions. In CHIKV nsP2 studies, docking has already been used to prioritize FDA‐approved compounds, and MD analyses based on root mean square deviation (RMSD), root mean square fluctuation (RMSF), and radius of gyration (Rg) have been applied to examine ligand stability and protein conformational behavior after binding [9]. This combined approach is particularly useful when the aim is not simply to identify the strongest single docking score but to find compounds that can engage two distinct viral proteins in a stable and biologically plausible manner. For a repurposing‐driven CHIKV project, such a framework is attractive because it balances mechanistic depth with translational practicality [9, 24].

Therefore, the main aim of this study is to screen FDA and internationally approved antimicrobials, veterinary therapeutics, and over‐the‐counter bioactive agents against CHIKV nsP2 and capsid, identify the compounds with the most favorable binding profiles for both proteins, and further validate the stability of the top‐ranked protein–ligand complexes through MD simulation in order to prioritize the most promising repurposable candidates for future experimental investigation. This study is novel in that it does not evaluate CHIKV drug candidates against a single viral protein, but instead adopts a dual‐target strategy focused on two functionally distinct and therapeutically relevant proteins, nsP2 and capsid, to identify FDA and internationally approved drugs with broader inhibitory potential. By integrating molecular docking with MD simulation, the study moves beyond static binding prediction and examines whether the selected antiviral compounds can form stable and biologically meaningful interactions with both targets under dynamic conditions. Such an approach is especially important for CHIKV, where no specific antiviral therapy has yet been approved and where simultaneous interference with viral replication and viral assembly may offer a stronger therapeutic rationale than single‐target inhibition.

## 2. Methodology

### 2.1. Protein Preparation

3D structure of CHIKV nsP2 protease (PDB ID: 3TRK) and capsid protein (PDB ID: 5H23) were downloaded in PDB format from RCSB Protein Data Bank (https://www.rcsb.org). The resolutions of 3TRK and 5H23 are 2.40 and 2.02 Å, respectively. Both structures were checked for completeness prior to docking. In 3TRK, three residues—Ser1003 (an N‐terminal expression‐tag residue) and Val1325/Gly1326 (the terminal C‐terminal residues)—were unresolved in the crystal structure. In 5H23, five N‐terminal residues (Cys106‐Glu110) were unresolved in each of the two chains (A and B). As these gaps are confined to disordered termini outside the nsP2 catalytic site and the capsid hydrophobic ligand‐binding pocket, respectively, no structural reconstruction was performed, and both structures were used as retrieved from the PDB for molecular docking. Structural cleanup was conducted prior to docking. All crystallographic water molecules, cocrystallized ligands, metal ions, and nonstructural buffer/crystallization additives (e.g., glycerol and sulfate ions) were removed using BIOVIA Discovery Studio Visualizer (Version 21.1.0.20298). Using AutoDockTools (Version 1.5.7), polar hydrogens and Kollman charges were added to the proteins. AD4 atom type was assigned, and the proteins were saved in pdbqt format. The docking regions of CHIKV nsP2 protease and capsid protein were defined in BIOVIA Discovery Studio using the cocrystallized ligand as the reference for native pocket identification. This ligand‐centered strategy was adopted to ensure that docking was performed within the experimentally resolved binding region rather than an arbitrary surface cavity.

### 2.2. Ligand Preparation

A screening set of 202 antimicrobial compounds was retrieved from the PubChem database. The dataset primarily consists of US FDA–approved small‐molecule drugs, supplemented by globally or veterinarily approved antimicrobials and bioactive reference molecules to maximize chemical diversity in targeting CHIKV proteins [9]. The 3D structures of the drugs were downloaded in SDF file format from PubChem (https://pubchem.ncbi.nlm.nih.gov). If 3D structure of a drug was not available in PubChem, then the 2D structure was retrieved from PubChem and converted into 3D structure using Avogadro software (Version 1.2.0). Protonation states of the ligands corresponding to physiological pH (pH 7.4) were assigned during polar hydrogen addition using AutoDockTools. Subsequently, nonpolar hydrogens were merged, Gasteiger partial charges were calculated, and torsions were assigned before saving the ligand structures in pdbqt format for molecular docking.

### 2.3. Molecular Docking

The selected ligands were docked against nsP2 protease and capsid protein using AutoDock Vina embedded within PyRx (Version 0.8), with the exhaustiveness parameter retained at its default value of 8 for all 202 ligand–protein combinations screened. The resulting pocket‐center coordinates were *x* = 3.5850, *y* = 32.3515, and *z* = 18.7099 for nsP2 protease, and *x* = 3.5801, *y* = −1.5593, and *z* = −17.8203 for the capsid protein. The grid box dimensions were set to $25.025.025.0\times \times \;\overset{{}^{\circ}}{\text{A}}$ along the *x*, *y*, and *z* axes to cover the entire binding cavity and allow full conformational and rotational search space for the docked ligands. For nsP2 protease, this search volume fully encompasses the catalytic dyad (CYS1013 and His1083) as well as the key neighboring substrate‐binding subpocket residues (TYR1079, TRP1084, ALA1046, and LYS1091), ensuring unrestricted sampling of the functional active site cleft. Two reference ligands were docked against their respective targets using the identical protocol. E‐64, an irreversible cysteine protease inhibitor previously reported to form a stable docked complex with CHIKV nsP2 protease, was docked against nsP2 protease and ethyl 3‐aminobenzoate (EAB), a ligand with experimentally confirmed nanomolar affinity for the capsid hydrophobic pocket, was docked against the capsid protein [9, 20]. The drugs were ranked based on the lowest binding energies. Protein–ligand interactions of the best two drugs were analyzed using BIOVIA Discovery Studio to reveal the noncovalent interactions and the key amino acid residues of the proteins involved for binding.

### 2.4. MD Simulation

To evaluate the dynamic stability of the selected protein–ligand complexes, 100‐ns MD simulations were performed for the best two complexes for each protein using the OpenMM simulation engine integrated within the Google Colaboratory environment framework described by Arantes et al. [25, 26]. Protonation states of the ligands were adjusted at pH 7.4 prior to topology generation. Ligand parameters were assigned using General AMBER Force Field 2 (GAFF2) [27]. The protein topologies were generated using the AMBER ff19SB force field, and the systems were solvated with the OPC explicit water model in a periodic simulation box with a 15‐Å buffer distance from the solute to the box edge. Net system charges were neutralized by adding counterions (N^+^ or Cl^−^), and additional NaCl was added to establish a physiological salt concentration of 0.15 M [28]. Energy minimization was performed for 20,000 steps using the L‐BFGS algorithm to eliminate steric overlaps. System equilibration was conducted for 5 ns under the isothermal‐isobaric (NPT) ensemble at 298.15 K and 1.0 atm using a 2.0‐fs integration time step. Temperature was maintained using a Langevin thermostat with a collision frequency of 1.0 ps^−1^), and pressure was regulated using a Monte Carlo barostat updated every 25 steps. Positional harmonic restraints (10.0 kcal/mol/AA^2^) were applied to all solute heavy atoms during equilibration. Covalent bonds involving hydrogen atoms were constrained using the SHAKE/HBonds algorithm. Long‐range electrostatic interactions were computed using the particle mesh Ewald (PME) method with a real‐space nonbonded cutoff distance of 10.0 Å.

Following equilibration, all positional restraints were completely released, and an unrestrained 100‐ns production simulation was carried out under the same NPT ensemble conditions (298.15 K and 1.0 atm) with a 2.0‐fs time step. Trajectory coordinates were saved every 100 ps, yielding 1000 frames per simulation for posttrajectory analysis. The resulting trajectories were analyzed in terms of RMSD, RMSF, and Rg to assess conformational stability and flexibility of the complexes. In addition, nonbonded interaction energy profiles comprising van der Waals and electrostatic energy components were extracted along the 100‐ns trajectories using PyTraj (v2.0.5) to monitor the noncovalent energetic persistence of the protein–ligand complexes over time [29].

## 3. Results

### 3.1. Molecular Docking

Docking analysis against CHIKV nsP2 protease identified several compounds with favorable docking scores within the selected binding pocket (Table 1). Among the screened compounds, bictegravir showed the lowest docking score (−9.5 kcal/mol), followed by simeprevir (−9.3 kcal/mol) and glecaprevir (−9.2 kcal/mol). In total, seven compounds produced docking scores of ≤ −9.0 kcal/mol, namely bictegravir, simeprevir, glecaprevir, grazoprevir, rifapentine, saquinavir, and ledipasvir. Paritaprevir, quinupristin, and velpatasvir also showed favorable binding, each with a docking score of −8.8 kcal/mol. The reference inhibitor E‐64 docked under the same protocol for comparison yielded a docking score of −6.1 kcal/mol, which is weaker than all 10 top‐ranked compounds in Table 1.

**Table 1:** Top‐ranked FDA and internationally approved drugs against CHIKV nsP2 protease based on docking score.

Sl. No.	Name	PubChem CID	Binding energy (kcal/mol)
1	E‐64	123985	−6.1
2	Bictegravir	90311989	−9.5
3	Simeprevir	24873435	−9.3
4	Glecaprevir	66828839	−9.2
5	Grazoprevir	44603531	−9.1
6	Rifapentine	135403821	−9.1
7	Saquinavir	441243	−9.1
8	Ledipasvir	67505836	−9.0
9	Paritaprevir	45110509	−8.8
10	Quinupristin	5388937	−8.8
11	Velpatasvir	67683363	−8.8

Docking against the CHIKV capsid protein also revealed several compounds with favorable predicted binding affinities (Table 2). Trovafloxacin showed the lowest docking score (−8.8 kcal/mol), followed by oxacillin (−8.7 kcal/mol) and paritaprevir (−8.6 kcal/mol). Other highly ranked compounds included glecaprevir and voxilaprevir (each −8.5 kcal/mol), elvitegravir (−8.3 kcal/mol), oxolinic acid (−8.1 kcal/mol), norfloxacin and tecovirimat (each −8.0 kcal/mol), and bictegravir (−7.9 kcal/mol). The reference ligand EAB scored −6.0 kcal/mol under the same protocol, weaker than all 10 top‐ranked capsid‐binding compounds in Table 2.

**Table 2:** Top‐ranked FDA and internationally approved drugs against CHIKV capsid protein based on docking score.

Sl. No.	Name	PubChem CID	Binding energy (kcal/mol)
1	Ethyl 3‐aminobenzoate	11400	−6.0
2	Trovafloxacin	62959	−8.8
3	Oxacillin	6196	−8.7
4	Paritaprevir	45110509	−8.6
5	Glecaprevir	66828839	−8.5
6	Voxilaprevir	89921642	−8.5
7	Elvitegravir	5277135	−8.3
8	Oxolinic Acid	4628	−8.1
9	Norfloxacin	4539	−8.0
10	Tecovirimat	16124688	−8.0
11	Bictegravir	90311989	−7.9

As theTop 3 docking hits were not common to both CHIKV nsP2 protease and capsid protein, the two highest ranked compounds for each target were selected for subsequent MD simulation. This approach enabled target‐wise assessment of the dynamic stability of the most favorable docked complexes. The docking scores of all the compounds are available in Table S1.

### 3.2. Nonbond Interactions

The interaction profiles of the top‐ranked docked complexes are presented in Figures 1, 2, 3, and 4 and summarized in Table 3. The selected compounds interacted with their respective CHIKV targets through multiple noncovalent forces, including hydrogen bonding, hydrophobic interactions, halogen interactions, electrostatic interactions, and *π*‐associated contacts. These interactions support the favorable docking scores observed for the selected ligands and suggest stable accommodation of the compounds within the predicted binding pockets.

**Figure 1:**
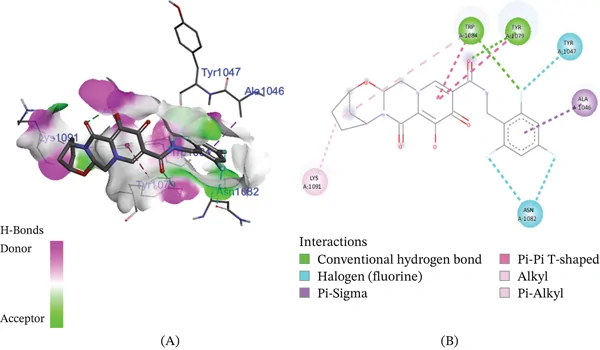
Predicted (A) 3D and (B) 2D nonbond interactions of bictegravir with nsP2.

**Figure 2:**
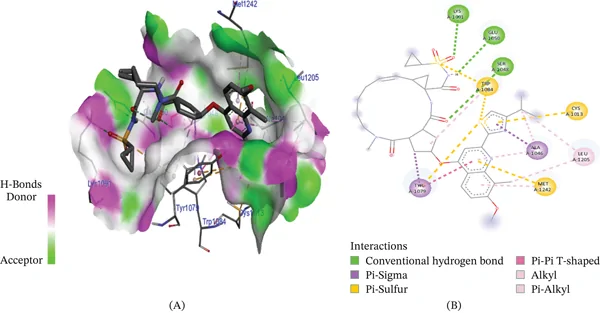
Predicted (A) 3D and (B) 2D nonbond interactions of simeprevir with nsP2.

**Figure 3:**
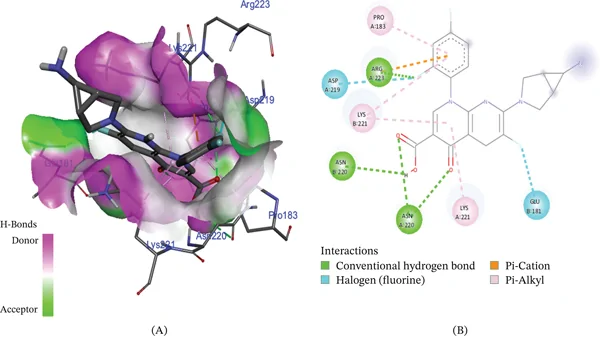
Predicted (A) 3D and (B) 2D nonbond interactions of trovafloxacin with capsid.

**Figure 4:**
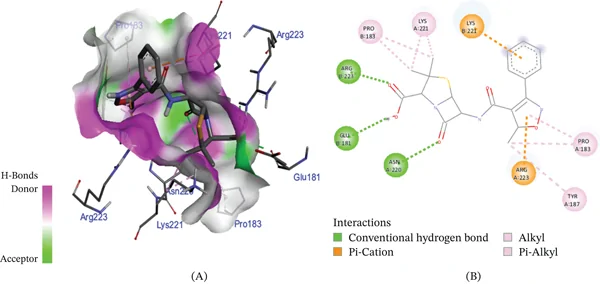
Predicted (A) 3D and (B) 2D nonbond interactions of oxacillin with capsid.

**Table 3:** Predicted noncovalent interaction profile of the top docked compounds with CHIKV nsP2 protease and capsid protein, showing bond categories, interaction types, and interacting amino acid residues.

Drug	Target	Bond category	Type	Amino acids
Bictegravir	nsP2	Hydrogen bond	Conventional hydrogen bond	TYR1079, TRP1084
		Halogen	Halogen (fluorine)	TYR1047, ASN1082
		Hydrophobic	Pi–sigma	ALA1046
			Pi–pi T‐shaped	TYR1079, TRP1084
			Alkyl	LYS1091
			Pi–alkyl	TRP1084

Simeprevir	nsP2	Hydrogen bond	Conventional hydrogen bond	SER1048, LYS1091, GLU1050
		Hydrophobic	Pi–sigma	ALA1046, TYR1079
			Pi–pi T‐shaped	TYR1079
			Alkyl	ALA1046, LEU1205
			Pi–alkyl	TRP1084, ALA1046, MET1242
		Other	Pi–sulfur	CYS1013, MET1242, TRP1084, TYR1079

Trovafloxacin	Capsid	Hydrogen bond	Conventional hydrogen bond	ASN220, ARG223
		Halogen	Halogen (fluorine)	ARG223, ASP219, GLU181
		Electrostatic	Pi–cation	ARG223
		Hydrophobic	Pi–alkyl	LYS221, PRO183

Oxacillin	Capsid	Hydrogen bond	Conventional hydrogen bond	ASN220, ARG223, GLU181
		Electrostatic	Pi–cation	ARG223, LYS221
		Hydrophobic	Alkyl	LYS221, PRO183
			Pi–alkyl	TYR187, PRO183, LYS221

For the bictegravir–nsP2 protease complex (Figure 1), bictegravir was predicted to form conventional hydrogen bonds in the docked pose with TYR1079 and TRP1084. In addition, halogen interactions involving fluorine were observed with TYR1047 and ASN1082. The ligand was further stabilized by several hydrophobic contacts, including a *π*–sigma interaction with ALA1046, *π*–*π* T‐shaped interactions with TYR1079 and TRP1084, an alkyl interaction with LYS1091, and a *π*–alkyl interaction with TRP1084. The presence of both hydrogen bonding and multiple aromatic/hydrophobic interactions describes predicted contacts within the selected nsP2 docking region.

The simeprevir–nsP2 protease complex (Figure 2) also displayed an extensive interaction network. Simeprevir was predicted to form conventional hydrogen bonds with SER1048, LYS1091, and GLU1050. Hydrophobic stabilization was provided through *π*–sigma interactions with ALA1046 and TYR1079, a *π*–*π* T‐shaped interaction with TYR1079, alkyl interactions with ALA1046 and LEU1205, and *π*–alkyl interactions involving TRP1084, ALA1046, and MET1242. In addition, *π*–sulfur interactions were observed with CYS1013, MET1242, TRP1084, and TYR1079. These diverse interactions suggest that simeprevir occupied the nsP2 binding pocket through a broad range of noncovalent contacts.

For the trovafloxacin–capsid protein complex (Figure 3), the ligand was predicted to form conventional hydrogen bonds with ASN220 and ARG223. Fluorine‐mediated halogen interactions were observed with ARG223, ASP219, and GLU181. Trovafloxacin also showed a *π*–cation electrostatic interaction with ARG223, along with *π*–alkyl hydrophobic interactions involving LYS221 and PRO183. These interactions indicate that trovafloxacin was stabilized within the capsid binding region through a combination of polar, halogen, electrostatic, and hydrophobic contacts.

The oxacillin–capsid protein complex (Figure 4) showed conventional hydrogen bonding with ASN220, ARG223, and GLU181. Electrostatic *π*–cation interactions were formed with ARG223 and LYS221, whereas hydrophobic stabilization was supported by alkyl interactions with LYS221 and PRO183 and *π*–alkyl interactions with TYR187, PRO183, and LYS221. The involvement of residues such as ASN220, ARG223, GLU181, LYS221, and PRO183 predicts strong oxacillin within the capsid binding pocket.

These findings support the docking results and further justify the selection of bictegravir and simeprevir for nsP2 and trovafloxacin and oxacillin for capsid protein as candidates for subsequent MD simulation.

### 3.3. MD Simulation

Figure 5 presents the 100‐ns MD simulation of the bictegravir–nsP2 protease complex. As shown in Figure 5a, the total interaction energy remained negative throughout the simulation, generally fluctuating between approximately −50 and −30 kcal/mol, indicating persistent favorable protein–ligand interactions over the full simulation period. This interaction profile was dominated by van der Waals contributions, which remained consistently negative and were more pronounced than the electrostatic component, suggesting that hydrophobic and close‐range noncovalent contacts were major contributors to complex stabilization. The electrostatic interaction energy also remained predominantly negative, although of smaller magnitude, indicating an additional favorable contribution to ligand binding.

**Figure 5:**
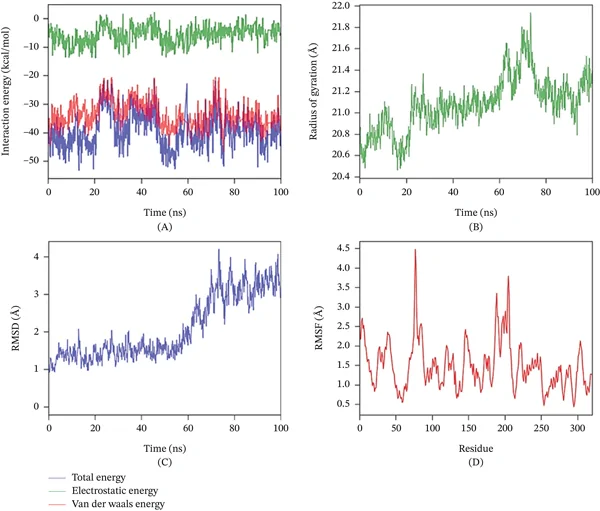
Molecular dynamics simulation of the bictegravir–nsP2 protease complex over 100 ns. (A) Total, electrostatic, and van der Waals interaction energies in kilocalories per mole; (B) radius of gyration (Rg) in angstrom; (C) root mean square deviation (RMSD) in angstrom; and (D) root mean square fluctuation (RMSF) in angstrom.

The structural compactness of the complex was further assessed by the Rg and RMSD profiles (Figure 5b,c). The Rg value remained within a relatively narrow range during the simulation, rising from about 20.6 to 20.7 Å at the beginning to approximately 21.2 Å by around 60 ns, followed by a transient increase to nearly 21.8 Å between 60 and 80 ns, and then partial relaxation toward the end of the trajectory. These modest fluctuations suggest that the nsP2 protease remained globally folded without evidence of major structural collapse. The RMSD profile showed an initial stabilization around 1.2–1.8 Å during the first half of the simulation, followed by a marked increase after approximately 60 ns, eventually reaching a higher plateau of about 3.0–3.8 Å. This pattern suggests that the complex underwent a conformational adjustment during the later stage of the simulation before reaching a new, relatively stable structural state rather than dissociating.

Residue‐wise flexibility was evaluated using the RMSF plot (Figure 5d). Most residues showed moderate fluctuations, whereas a limited number of regions displayed higher mobility, particularly around the peaks observed near residues ~75 and ~190–210. These localized increases in flexibility likely reflect loop or surface‐exposed regions of the protein rather than overall destabilization of the complex. The MD results suggest that the bictegravir–nsP2 protease complex maintained favorable interactions throughout the simulation, with van der Waals forces contributing prominently to binding stability despite a noticeable conformational rearrangement during the later phase of the trajectory.

Figure 6 summarizes the 100‐ns MD trajectory of the simeprevir–nsP2 protease complex. Unlike the bictegravir‐bound system, the interaction‐energy profile in Figure 6a was marked by several abrupt positive excursions, indicating a substantially more irregular energetic pattern across the simulation. Because these spikes were intermittent rather than sustained, they may reflect transient perturbations during the trajectory rather than continuous loss of association. However, this profile suggests that the simeprevir‐bound complex was energetically less uniform than the bictegravir‐bound complex. In contrast, the global structural descriptors indicated only moderate conformational variation. The Rg initially remained around 20.5–20.7 Å, increased gradually to approximately 21.1–21.4 Å during the middle portion of the simulation, and then declined toward the end of the run (Figure 6b), pointing to a temporary expansion followed by partial compaction of the protein structure. A comparable trend was observed in the RMSD trace (Figure 6c), which rose from nearly 1.0 Å at the start to around 1.5 Å by the early‐to‐middle phase, followed by a broader increase with peaks above 2.0 Å around 50–60 ns, after which the trajectory settled mainly within the 1.5–1.9‐Å range. This behavior is more consistent with conformational adjustment than with large‐scale destabilization. Residue‐level fluctuations were also limited overall, with most residues showing RMSF values below 1.5 Å, although a few localized peaks were evident, particularly around the 80–100 and 190–210 residue regions (Figure 6d). These data suggest that the simeprevir–nsP2 protease complex underwent noticeable but contained structural rearrangements during the simulation and retained an overall stable fold despite its more erratic interaction‐energy profile.

**Figure 6:**
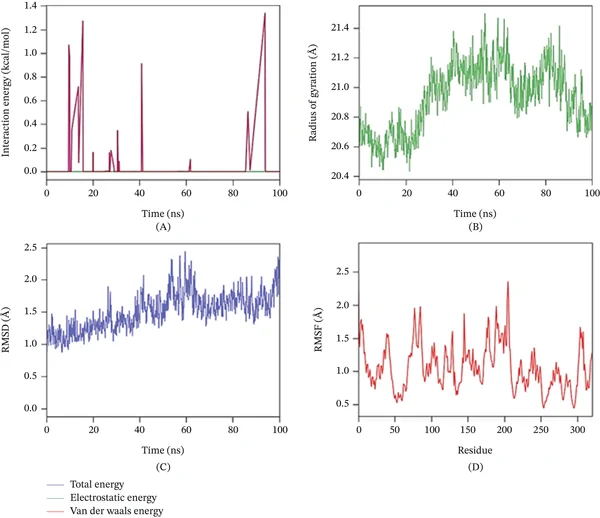
Molecular dynamics simulation of the simeprevir–nsP2 protease complex over 100 ns. (A) Total, electrostatic, and van der Waals interaction energies in kilocalories per mole; (B) radius of gyration (Rg) in angstrom; (C) root mean square deviation (RMSD) in angstrom; and (D) root mean square fluctuation (RMSF) in angstrom.

Figure 7 presents the 100‐ns MD simulation profile of the trovafloxacin–capsid protein complex. The interaction‐energy trace showed substantial short‐term fluctuations throughout the trajectory, particularly in the electrostatic component, whereas the van der Waals contribution remained comparatively smoother and slightly favorable across most of the simulation period (Figure 7a). Despite these oscillations, the total interaction energy stayed predominantly in the negative range, indicating that the complex maintained an overall favorable interaction pattern during the run. The Rg profile remained tightly distributed, largely between 22.5 and 23.0 Å, with only a mild downward tendency toward the later stage of the simulation (Figure 7b), suggesting preservation of the overall compactness of the protein structure. In contrast, the RMSD trace showed a gradual increase over time, beginning at approximately 1.1–1.4 Å, rising to around 1.8–2.0 Å during the first half of the trajectory, and reaching higher values of nearly 2.8–3.1 Å between 60 and 85 ns before ending at a slightly lower level (Figure 7c). This pattern suggests that the complex underwent progressive conformational adjustment during the simulation rather than abrupt structural disruption. Residue‐level flexibility was generally modest, although several localized peaks were observed in the RMSF profile, especially around residues ~40, ~150, and ~200 (Figure 7d), indicating the presence of a few more mobile segments within an otherwise stable protein framework. These findings suggest that the trovafloxacin–capsid protein complex retained global structural stability over 100 ns, although it exhibited greater conformational drift and localized flexibility than a highly rigid complex.

**Figure 7:**
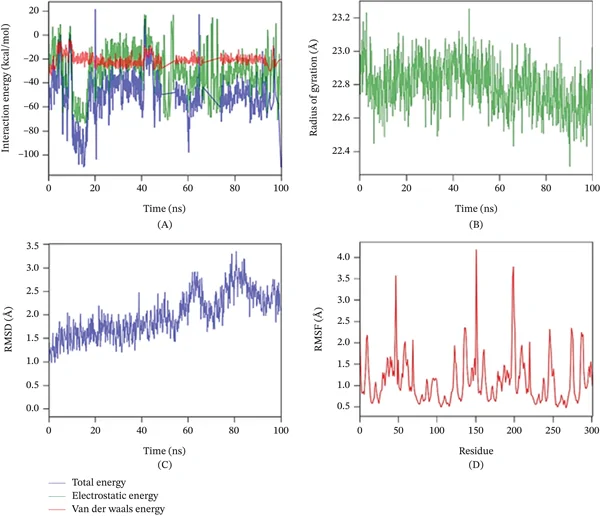
100‐ns MD simulation results of trovafloxacin–capsid protein complex. (A) Interaction energy in kilocalories per mole, (B) radius of gyration in angstrom, (C) RMSD in angstrom, and (D) RMSF in angstrom.

Figure 8 depicts the 100‐ns MD trajectory of the oxacillin–capsid protein complex. The interaction‐energy profile was comparatively noisy across the simulation, particularly for the electrostatic component, whereas the van der Waals term fluctuated within a narrower window (Figure 8a). Despite this variability, the overall interaction pattern remained favorable throughout the trajectory, supporting continued association of oxacillin with the capsid binding region during the simulation period. At the structural level, the complex showed no evidence of major expansion or collapse, as the Rg remained largely confined between approximately 22.4 and 23.2 Å (Figure 8b). The RMSD trace also stayed within a relatively limited range for most of the run, generally around 1.4–2.0 Å, with only a mild upward drift toward the final portion of the trajectory (Figure 8c), suggesting gradual structural adjustment rather than pronounced destabilization. Residue‐wise fluctuations were modest overall, with most positions showing low to moderate mobility and only a few localized peaks, the most prominent occurring around residue 145, alongside smaller flexible segments in the 180–220 region (Figure 8d). Collectively, these findings indicate that the oxacillin–capsid protein complex remained structurally well maintained over 100 ns, while retaining a somewhat dynamic interaction profile.

**Figure 8:**
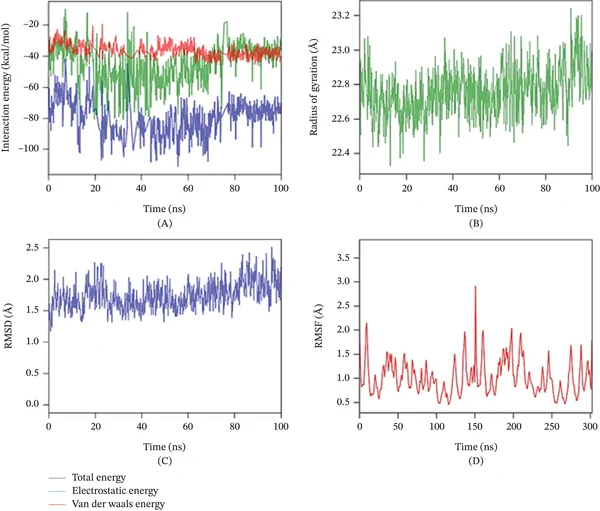
Molecular dynamics simulation of the oxacillin–capsid protein complex over 100 ns. (A) Total, electrostatic, and van der Waals interaction energies in kilocalories per mole; (B) radius of gyration (Rg) in angstrom; (C) root mean square deviation (RMSD) in angstrom; and (D) root mean square fluctuation (RMSF) in angstrom.

## 4. Discussion

The present study identified bictegravir and simeprevir as the strongest nsP2 protease docking hits, whereas trovafloxacin and oxacillin ranked highest against the CHIKV capsid target. The protein–ligand interaction profiles further support the docking‐based prioritization of the selected compounds. In the nsP2 complexes, bictegravir and simeprevir interacted with several residues within the predicted binding pocket through hydrogen bonding, hydrophobic contacts, *π*‐associated interactions, and, in the case of bictegravir, fluorine‐mediated halogen interactions. The involvement of residues such as TYR1079, TRP1084, ALA1046, and LYS1091 suggests that aromatic and hydrophobic contacts may contribute substantially to ligand stabilization within the nsP2 pocket. Bictegravir formed hydrogen bonds with TYR1079 and TRP1084 along with multiple *π*‐interactions, which may partly explain its favorable docking score and stable MD behavior. Simeprevir also formed several hydrogen‐bonding and hydrophobic interactions; however, its less uniform MD interaction‐energy profile indicates that the presence of multiple docking interactions alone may not always translate into superior dynamic stability.

Similarly, the capsid‐binding compounds showed interaction patterns consistent with their favorable docking scores. Trovafloxacin and oxacillin both interacted with key capsid residues such as ASN220, ARG223, LYS221, PRO183, and GLU181, suggesting that this region may provide a suitable binding environment for structurally diverse ligands. Trovafloxacin showed hydrogen bonding, halogen bonding, *π*–cation, and hydrophobic contacts, whereas oxacillin formed hydrogen bonds with ASN220, ARG223, and GLU181, along with *π*–cation and hydrophobic interactions involving ARG223, LYS221, PRO183, and TYR187. The stronger dynamic restraint observed for oxacillin despite its slightly weaker docking score emphasizes that favorable noncovalent contacts should be interpreted together with MD stability parameters rather than docking score alone. It should be noted that small differences between docking scores should be interpreted cautiously because docking scoring functions provide approximate estimates of binding affinity [30]. For example, the scores obtained for trovafloxacin (−8.8 kcal/mol) and oxacillin (−8.7 kcal/mol) are comparable, and their 0.1 kcal/mol difference does not establish stronger binding by trovafloxacin. Similarly, small score differences among the nsP2 candidates do not demonstrate differences in experimental affinity or antiviral potency. The reported rankings therefore represent numerical ordering for computational prioritization, with experimental measurements required to establish differences in binding affinity.

The subsequent 100‐ns simulations then suggested that bictegravir formed the more stable nsP2 complex, whereas oxacillin showed a more restrained capsid‐bound trajectory than trovafloxacin. These findings are relevant because CHIKV still lacks a specific approved antiviral treatment, and patient care remains largely supportive despite the fact that arthralgia can persist for months or even years [1, 31].

Taken together, these results show that docking rankings were not uniformly reproduced across the MD descriptors. For nsP2 protease, bictegravir achieved the most favorable docking score and maintained consistently negative interaction energies, but its complex exhibited a higher late‐stage RMSD than the simeprevir complex. Thus, the relative assessment of these compounds depended on the MD descriptor considered. For the capsid protein, trovafloxacin obtained the best docking score, whereas oxacillin, ranked second, displayed a more restrained RMSD profile over the 100‐ns simulation. Such differences do not necessarily indicate methodological error. Docking estimates the favorability of an initial binding pose, whereas MD examines subsequent conformational behavior in explicit solvent [32]. Conformational relaxation and rearrangement of protein–ligand or solvent interactions could contribute to these differences, although the underlying mechanisms cannot be established from RMSD and interaction‐energy profiles alone. These findings support considering docking scores alongside multiple MD descriptors when prioritizing candidates for experimental evaluation.

The nsP2 findings are biologically plausible. CHIKV nsP2 protease is required for cleavage of the viral nonstructural polyprotein, and this processing step is essential for viral replication, which is why nsP2 has been widely regarded as a high‐value antiviral target [16, 33]. The strong ranking of bictegravir, simeprevir, glecaprevir, grazoprevir, and paritaprevir in the present docking set is not random. Several of these molecules were originally developed to engage viral enzymes, especially protease‐centered systems, so their enrichment around the nsP2 pocket is consistent with the catalytic role of CHIKV nsP2 [33, 34]. Mechanistically, the binding interactions directly involve or lie adjacent to the catalytic machinery of the nsP2 cysteine protease. Specifically, CYS1013 (Cys478 in mature nsP2 numbering) is the catalytic nucleophile, whereas TRP1084 (Trp549) immediately follows the catalytic HIS1083 (His548) and contributes to substrate recognition. TYR1079 (Tyr544) forms part of the S3 substrate‐binding subsite [35]. In the present study, bictegravir formed predicted hydrogen bonds with TYR1079 and TRP1084, whereas simeprevir exhibited hydrophobic contacts with these residues and a *π*–sulfur interaction with CYS1013. These contacts support a plausible mechanism involving interference with substrate access or positioning during nonstructural polyprotein cleavage. However, the predicted interactions do not establish active‐site occlusion or functional inhibition, which require experimental confirmation.

Among the nsP2 hits, simeprevir is reported in recent structure‐based repurposing work as a strong binder of CHIKV nsP2. At the same time, the current data suggest that bictegravir may deserve closer attention. Although bictegravir is not a classical viral protease inhibitor, it achieved the lowest docking score against nsP2 in this study, and more importantly, maintained a more favorable MD profile than simeprevir, with persistently negative interaction energy and a more coherent structural trajectory overall. This difference matters because docking alone provides only a static estimate of pose favorability, whereas MD helps determine whether a docked complex remains geometrically and energetically reasonable over time. RMSD, interaction persistence, and related descriptors are widely used for that purpose, and native‐like poses tend to show more stable behavior during simulation than poor or decoy poses [36].

The contrast between bictegravir and simeprevir also illustrates a broader point about computational prioritization. In the present data, simeprevir retained an overall folded nsP2 complex but showed a more irregular interaction‐energy pattern and a more visibly perturbed trajectory than bictegravir. This suggests that the rank order generated by docking can change once the system is allowed to move in explicit solvent. That is not a weakness of the study. Rather, it is one of the main reasons to combine docking with MD. For flexible proteins or protein pockets that adapt to ligand shape, postdocking dynamic assessment often provides a more realistic basis for selecting leads for experimental work [37]. In practical terms, the nsP2 part of the present study therefore points more strongly toward bictegravir than toward simeprevir, even though both should still be viewed as computational leads rather than confirmed inhibitors.

CHIKV capsid is more than a passive structural protein. It packages the viral RNA and contributes directly to nucleocapsid assembly and virus budding through interaction with the E2 cytoplasmic tail. A conserved hydrophobic pocket in the capsid protease domain has been structurally defined and proposed as a druggable site [4, 20]. However, the capsid‐interacting residues identified in this study (GLU181, PRO183, ASP219, ASN220, LYS221, and ARG223) do not correspond to the E2‐cytoplasmic‐tail‐binding hydrophobic pocket previously described for CHIKV capsid protein (Val130, Gly131, Val134, Met135, Trp245, and Val250) [20]. Instead, this residue cluster lies in the vicinity of the capsid protein′s own autoproteolytic machinery, including Ser213, the catalytic serine of its serine‐protease triad, and Ile227, a residue reported to lie spatially close to the catalytic triad and to be required for efficient proteolytic activity [38, 39]. This raises the possibility that the compounds identified here may interfere with capsid autoproteolytic processing or its substrate‐specificity pocket, a mechanistically distinct but equally essential step in structural polyprotein maturation, rather than, or in addition to, disrupting E2–capsid coupling. Structural validation, such as targeted mutagenesis or cocrystallography of these residues would help confirm this alternative mode of action.

An interesting feature of the capsid results is the chemical diversity of the top‐ranked compounds. Unlike the nsP2 list, which was enriched with antiviral agents and viral enzyme inhibitors, the leading capsid hits included antibacterial drugs such as trovafloxacin and oxacillin. It does not mean that these compounds are established anti‐CHIKV agents. Instead, it suggests that the capsid may accommodate a broader range of scaffolds than the catalytic nsP2 pocket. That interpretation is consistent with previous structural work showing that relatively small and chemically distinct ligands can occupy this region [20]. The capsid pocket may be less class‐restricted than nsP2, which makes it attractive for repurposing screens but also increases the need for experimental confirmation.

The MD comparison between oxacillin and trovafloxacin is especially informative. Trovafloxacin produced the best capsid docking score, yet its trajectory showed greater conformational drift over time, with a progressive RMSD increase and several localized flexible regions. Oxacillin, despite ranking second in docking, displayed a narrower RMSD range and a more restrained overall structural pattern. This again shows why docking score should not be used as the sole criterion for lead nomination. A top‐scoring ligand may still prove dynamically less persuasive than a slightly lower ranked alternative once protein motion, solvent effects, and pocket adaptation are considered [36]. On the basis of the present data alone, oxacillin appears to be the more stable capsid‐associated complex, whereas trovafloxacin appears to be the more flexible one.

Another valuable observation is the recurrence of a few compounds across both targets. Paritaprevir, glecaprevir, and bictegravir appeared in the upper range of both docking lists. Although only bictegravir was carried forward into MD among these shared hits, this overlap is still important because compounds with multitarget behavior may offer practical advantages in antiviral development. Polypharmacology‐guided antiviral strategies have been proposed as one way to improve antiviral effect and, in some settings, to reduce the risk of rapid resistance selection that often limits single‐target monotherapies [40–42]. From that perspective, the shared appearance of paritaprevir, glecaprevir, and bictegravir makes them attractive candidates for a second‐round prioritization, particularly if future work includes longer simulations, per‐residue energy decomposition, and wet‐lab assays.

The most promising target‐complementary drug combination emerging from this study appears to be bictegravir plus oxacillin. In the present analysis, bictegravir showed the strongest docking performance against nsP2 protease and retained the more favorable nsP2‐bound dynamic profile, whereas oxacillin showed the more restrained and structurally better maintained trajectory against the capsid target than trovafloxacin. Because nsP2 protease is essential for nonstructural polyprotein processing and viral replication, although the conserved capsid hydrophobic pocket contributes to E2 interaction, nucleocapsid assembly, and virus budding, simultaneous inhibition of these two proteins could, in principle, interfere with two distinct stages of the CHIKV life cycle. This type of dual‐target strategy is also biologically attractive because antiviral combinations that act at different stages of the viral cycle can provide broader antiviral pressure and may offer advantages over a single‐target approach. For these reasons, bictegravir plus oxacillin may be considered the leading candidate combination from the current dataset, whereas shared binders such as paritaprevir and glecaprevir remain important secondary candidates for future evaluation as broader dual‐target leads.

The present findings can be contextualized against earlier computational and experimental studies targeting CHIKV nsP2 and capsid. Kumar et al. [9] used docking and MD simulations to prioritize ribostamycin sulfate and E‐64 as potential nsP2 inhibitors. Our study similarly prioritized bictegravir against nsP2, with a docking score of −9.5 kcal/mol and persistently negative interaction energies, although its later RMSD increase indicates conformational adjustment. Kalam et al. [34] computationally prioritized simeprevir and voxilaprevir against nsP2 and subsequently evaluated their antiviral activity in vitro. The high ranking of simeprevir in our screen (−9.3 kcal/mol) shows qualitative agreement with that study, whereas bictegravir ranked ahead within our dataset. For capsid, Sharma et al. [21] demonstrated that picolinic acid binds the conserved hydrophobic pocket and inhibits CHIKV replication in infected cells, providing experimental support for investigating this protein as an antiviral target. Against this background, oxacillin′s docking score of −8.7 kcal/mol and comparatively restrained RMSD profile provide a computational rationale for investigating another chemically distinct compound against capsid. However, these findings do not establish that bictegravir or oxacillin is more potent than previously reported candidates. The contribution of the present study is their prioritization for further investigation across two complementary viral targets, with biochemical and cell‐based validation required to determine whether the predicted interactions translate into antiviral activity.

Bictegravir was developed as an HIV‐1 integrase strand transfer inhibitor [43]. Its proposed activity against CHIKV nsP2 therefore requires independent validation. In our docking model, hydrogen bonds with TYR1079 and TRP1084, residues associated with substrate recognition, provide a structural rationale for possible interference with substrate binding [35]. However, these predicted contacts and the MD results do not establish selective nsP2 inhibition or exclude nonspecific binding, effects on host proteins, or cytotoxicity. Biochemical inhibition and cellular target‐engagement studies, together with antiviral testing and matched cell‐viability measurements, are needed to determine whether any antiviral effect occurs at noncytotoxic concentrations and is attributable to nsP2 inhibition. Bictegravir should therefore be regarded as a computationally prioritized candidate whose mechanism, selectivity, and safety in the CHIKV context remain unresolved.

This study also has important limitations. First, docking scores and MD‐derived stability patterns are useful for ranking compounds, but they do not prove biochemical inhibition or antiviral efficacy. Experimental validation remains essential. Second, the current MD analysis covered only four complexes and a 100‐ns timescale. That duration is reasonable for an initial comparison, but more flexible systems or induced‐fit effects may require longer trajectories for firmer conclusions. Finally, repurposing candidates such as oxacillin and trovafloxacin raise practical translational questions because target engagement alone does not address whether antiviral exposure can be reached safely in infected tissues. Future research should include biochemical target validation, cell‐based antiviral testing, and more detailed free‐energy or contact analyses before any stronger therapeutic claims are made.

## 5. Conclusion

In conclusion, this study identified several FDA and internationally approved therapeutics with favorable predicted binding against two therapeutically relevant CHIKV targets, namely nsP2 protease and the capsid protein. Molecular docking showed that bictegravir and simeprevir were the leading candidates against nsP2 protease, whereas trovafloxacin and oxacillin emerged as the top‐ranked compounds against the capsid target. These findings suggest that both viral proteins contain binding regions that can accommodate structurally diverse approved drugs and may therefore be useful targets for repurposing‐based antiviral discovery. The MD results helped refine this initial ranking by providing a dynamic view of protein–ligand behavior over time. Among the nsP2 complexes, bictegravir showed a more favorable predicted overall stability profile, with persistent negative interaction energy and acceptable structural compactness despite some late conformational adjustment. Simeprevir also remained associated with the target but displayed a more irregular energetic pattern. For the capsid target, both oxacillin and trovafloxacin maintained overall structural stability during the 100‐ns simulations, although oxacillin showed a comparatively more predicted restrained trajectory, whereas trovafloxacin exhibited greater conformational drift. Taken together, these results suggest that bictegravir may be the more promising nsP2‐directed candidate, whereas oxacillin may represent the more stable capsid‐directed lead.

From a broader perspective, the present work supports a dual‐target screening strategy for CHIKV drug repurposing. Because nsP2 protease is essential for viral polyprotein processing and replication, and the capsid protein contributes to nucleocapsid assembly and viral particle formation, simultaneous targeting of both proteins may offer a more effective antiviral approach than single‐target inhibition alone. On the basis of the current dataset, bictegravir plus oxacillin appears to be the most promising target‐complementary combination for future study. Nevertheless, these findings remain computational and should be interpreted cautiously. Further work should include longer simulations, binding free‐energy refinement, enzymatic assays, and cell‐based antiviral studies to confirm whether the prioritized compounds can produce meaningful anti‐CHIKV activity in biological systems. If experimentally validated, the prioritized compounds could provide new starting points for CHIKV antiviral development, supporting broader efforts to expand therapeutic options for emerging and re‐emerging arboviral infections of global health concern.

## Author Contributions

Asef Raj: conceptualization, supervision, software, data analysis, writing—original draft and final draft, writing—review and editing, and visualization; Humair Bin Md Omer: software, data analysis, writing—final draft, and visualization; Mim Deb Roy: software, data analysis, and writing—final draft; Aisha Siddika: software, data analysis, and writing—final draft; Sabrina Sharmin: writing—review and editing.

## Funding

No funding was received for this manuscript.

## Disclosure

The authors take full responsibility for the accuracy, originality, and integrity of the final manuscript.

## Ethics Statement

The authors have nothing to report.

## Consent

The authors have nothing to report.

## Conflicts of Interest

The authors declare no conflicts of interest.

## Supporting information


**Supporting Information** Additional supporting information can be found online in the Supporting Information section. Docking scores of 202 FDA and internationally approved drugs against CHIKV nsp2 protease and capsid protein (Table S1).

## Data Availability

The data that support the findings of this study are available from the corresponding author upon reasonable request.

## References

[1] World Health Organization (WHO) , Chikungunya Epidemiology Update - June 2025, 2025, World Health Organization (WHO), https://www.who.int/publications/m/item/chikungunya-epidemiology-update-june-2025.

[2] US Centers for Disease Control and Prevention , Chikungunya, 2026, US Centers for Disease Control and Prevention, https://www.cdc.gov/yellow-book/hcp/travel-associated-infections-diseases/chikungunya.html.

[3] US Centers for Disease Control and Prevention , Symptoms, Diagnosis, & Treatment, 2024, US Centers for Disease Control and Prevention, https://www.cdc.gov/chikungunya/symptoms-diagnosis-treatment/index.html.

[4] Martelossi-Cebinelli G. , Carneiro J. A. , Yaekashi K. M. , Bertozzi M. M. , Bianchini B. H. S. , Rasquel-Oliveira F. S. , Zanluca C. , dos Santos C. N. D. , Arredondo R. , Blackburn T. A. , Casagrande R. , and Verri W. A. , A Review of the Biology of Chikungunya Virus Highlighting the Development of Current Novel Therapeutic and Prevention Approaches, Pathogens. (2025) 14, no. 10, 10.3390/pathogens14101047, 41156657. PMC12567008 41156657

[5] Kasabe B. , Ahire G. , Patil P. , Punekar M. , Davuluri K. S. , Kakade M. , Alagarasu K. , Parashar D. , and Cherian S. , Drug Repurposing Approach Against Chikungunya Virus: An In Vitro and In Silico Study, Frontiers in Cellular and Infection Microbiology. (2023) 13, 1132538, 10.3389/fcimb.2023.1132538, 37180434. PMC10174255 37180434

[6] Ferreira A. C. , Reis P. A. , de Freitas C. S. , Sacramento C. Q. , Villas Bôas Hoelz L. , Bastos M. M. , Mattos M. , Rocha N. , Gomes de Azevedo Quintanilha I. , da Silva Gouveia Pedrosa C. , Rocha Quintino Souza L. , Correia Loiola E. , Trindade P. , Rangel Vieira Y. , Barbosa-Lima G. , de Castro Faria Neto H. C. , Boechat N. , Rehen S. K. , Brüning K. , Bozza F. A. , Bozza P. T. , and Souza T. M. L. , Beyond Members of the Flaviviridae Family, Sofosbuvir Also Inhibits Chikungunya Virus Replication, Antimicrobial Agents and Chemotherapy. (2019) 63, no. 2, e01389-18, 10.1128/AAC.01389-18. PMC6355571 30455237

[7] Battisti V. , Urban E. , and Langer T. , Antivirals Against the Chikungunya Virus, Viruses. (2021) 13, no. 7, 10.3390/v13071307, 34372513. PMC8310245 34372513

[8] Adamson C. S. , Chibale K. , Goss R. J. M. , Jaspars M. , Newman D. J. , and Dorrington R. A. , Antiviral Drug Discovery: Preparing for the Next Pandemic, Chemical Society Reviews. (2021) 50, no. 6, 3647–3655, 10.1039/D0CS01118E, 33524090. 33524090

[9] Kumar P. , Kumar D. , and Giri R. , Targeting the nsp2 Cysteine Protease of Chikungunya Virus Using FDA Approved Library and Selected Cysteine Protease Inhibitors, Pathogens. (2019) 8, no. 3, 10.3390/pathogens8030128, 31443266. PMC6789655 31443266

[10] Kiser L. M. , Sokoloski K. J. , and Hardy R. W. , Interactions Between Capsid and Viral RNA Regulate Chikungunya Virus Translation in a Host-Specific Manner, Virology. (2021) 560, 34–42, 10.1016/j.virol.2021.04.009, 34023723. PMC8206026 34023723

[11] Verwimp S. , Wagoner J. , Arenas E. G. , De Coninck L. , Abdelnabi R. , Hyde J. L. , Schiffer J. T. , White J. M. , Matthijnssens J. , Neyts J. , Polyak S. J. , and Delang L. , Combinations of Approved Oral Nucleoside Analogues Confer Potent Suppression of Alphaviruses *In Vitro* and *In Vivo* , Antiviral Research. (2025) 239, 106186, 10.1016/j.antiviral.2025.106186, 40379030. 40379030

[12] Law Y.-S. , Utt A. , Tan Y. B. , Zheng J. , Wang S. , Chen M. W. , Griffin P. R. , Merits A. , and Luo D. , Structural Insights Into RNA Recognition by the Chikungunya Virus nsP2 Helicase, Proceedings of the National Academy of Sciences. (2019) 116, no. 19, 9558–9567, 10.1073/pnas.1900656116, 31000599. PMC6511008 31000599

[13] Bakar F. A. and Ng L. F. P. , Nonstructural Proteins of Alphavirus—Potential Targets for Drug Development, Viruses. (2018) 10, no. 2, 10.3390/v10020071. PMC5850378 29425115

[14] Wong K. Z. and Chu J. J. H. , The Interplay of Viral and Host Factors in Chikungunya Virus Infection: Targets for Antiviral Strategies, Viruses. (2018) 10, no. 6, 10.3390/v10060294, 29849008. PMC6024654 29849008

[15] Raj S. , Irfan M. , and Ranjith-Kumar C. T. , Chikungunya Virus Non-Structural Protein 2 (nsP2) Inhibits RIG-I and TLR-Mediated Immune Response, Virology. (2026) 616, 110777, 10.1016/j.virol.2025.110777, 41447922. 41447922

[16] Merten E. M. , Sears J. D. , Leisner T. M. , Hardy P. B. , Ghoshal A. , Hossain M. A. , Asressu K. H. , Brown P. J. , Tse E. G. , Stashko M. A. , Li K. , Norris-Drouin J. L. , Herring L. E. , Mordant A. L. , Webb T. S. , Mills C. A. , Barker N. K. , Streblow Z. J. , Perveen S. , Arrowsmith C. H. , Couñago R. M. , Arnold J. J. , Cameron C. E. , Streblow D. N. , Moorman N. J. , Heise M. T. , Willson T. M. , Popov K. I. , and Pearce K. H. , Identification of a Cell-Active Chikungunya Virus nsP2 Protease Inhibitor Using a Covalent Fragment-Based Screening Approach, Proceedings of the National Academy of Sciences. (2024) 121, no. 42, e2409166121, 10.1073/pnas.2409166121, 39388272. PMC11494320 39388272

[17] Sharma K. B. , Subramani C. , Ganesh K. , Sharma A. , Basu B. , Balyan S. , Sharma G. , Ka S. , Deb A. , Srivastava M. , Chugh S. , Sehrawat S. , Bharadwaj K. , Rout A. , Sahoo P. K. , Saurav S. , Motiani R. K. , Singh R. , Jain D. , Asthana S. , Wadhwa R. , and Vrati S. , Withaferin A Inhibits Chikungunya Virus nsP2 Protease and Shows Antiviral Activity in the Cell Culture and Mouse Model of Virus Infection, PLoS Pathogens. (2024) 20, no. 12, e1012816, 10.1371/journal.ppat.1012816, 39775571. PMC11723598 39775571

[18] Bhardwaj K. , Ranjith-Kumar C. T. , Guchhait P. , and Vrati S. , Current Perspectives on Alphavirus Encapsidation, Assembly and Budding, Frontiers in Cellular and Infection Microbiology. (2026) 15, 1719868, 10.3389/fcimb.2025.1719868, 41561097. PMC12812552 41561097

[19] Sun S. , Xiang Y. , Akahata W. , Holdaway H. , Pal P. , Zhang X. , Diamond M. S. , Nabel G. J. , and Rossmann M. G. , Structural Analyses at Pseudo Atomic Resolution of Chikungunya Virus and Antibodies Show Mechanisms of Neutralization, eLife. (2013) 2, e00435, 10.7554/eLife.00435, 23577234. PMC3614025 23577234

[20] Sharma R. , Kesari P. , Kumar P. , and Tomar S. , Structure-Function Insights Into Chikungunya Virus Capsid Protein: Small Molecules Targeting Capsid Hydrophobic Pocket, Virology. (2018) 515, 223–234, 10.1016/j.virol.2017.12.020, 29306785. 29306785

[21] Sharma R. , Fatma B. , Saha A. , Bajpai S. , Sistla S. , Dash P. K. , Parida M. , Kumar P. , and Tomar S. , Inhibition of Chikungunya Virus by Picolinate That Targets Viral Capsid Protein, Virology. (2016) 498, 265–276, 10.1016/j.virol.2016.08.029, 27614702. 27614702

[22] Thomas S. , Rai J. , John L. , Schaefer S. , Pützer B. M. , and Herchenröder O. , Chikungunya Virus Capsid Protein Contains Nuclear Import and Export Signals, Virology Journal. (2013) 10, no. 1, 10.1186/1743-422X-10-269, 23984714. PMC3765696 23984714

[23] Taylor A. , Liu X. , Zaid A. , Goh L. Y. H. , Hobson-Peters J. , Hall R. A. , Merits A. , Mahalingam S. , Diamond M. , Weaver S. , and Morrison T. , Mutation of the N-Terminal Region of Chikungunya Virus Capsid Protein: Implications for Vaccine Design, mBio. (2017) 8, no. 1, e01970-16, 10.1128/mBio.01970-16. PMC5358915 28223458

[24] Rabelo V. W. , Sanchez-Nuñez M. L. , Corrêa-Amorim L. S. , Kuhn R. J. , Abreu P. A. , and Paixão I. C. N. P. , In Silico Drug Repurposing Uncovered the Antiviral Potential of the Antiparasitic Drug Oxibendazole Against the Chikungunya Virus, ACS Omega. (2024) 9, no. 25, 27632–27642, 10.1021/acsomega.4c03417, 38947813. PMC11209700 38947813

[25] Arantes P. R. , Polêto M. D. , Pedebos C. , and Ligabue-Braun R. , Making It Rain: Cloud-Based Molecular Simulations for Everyone, Journal of Chemical Information and Modeling. (2021) 61, no. 10, 4852–4856, 10.1021/acs.jcim.1c00998, 34595915. 34595915

[26] Eastman P. , Galvelis R. , Peláez R. P. , Abreu C. R. A. , Farr S. E. , Gallicchio E. , Gorenko A. , Henry M. M. , Hu F. , Huang J. , Krämer A. , Michel J. , Mitchell J. A. , Pande V. S. , Rodrigues J. P. , Rodriguez-Guerra J. , Simmonett A. C. , Singh S. , Swails J. , Turner P. , Wang Y. , Zhang I. , Chodera J. D. , De Fabritiis G. , and Markland T. E. , OpenMM 8: Molecular Dynamics Simulation With Machine Learning Potentials, Journal of Physical Chemistry B. (2024) 128, no. 1, 109–116, 10.1021/acs.jpcb.3c06662, 38154096. PMC10846090 38154096

[27] He X. , Man V. H. , Yang W. , Lee T.-S. , and Wang J. , A Fast and High-Quality Charge Model for the Next Generation General AMBER Force Field, Journal of Chemical Physics. (2020) 153, no. 11, 114502, 10.1063/5.0019056, 32962378. PMC7728379 32962378

[28] Tian C. , Kasavajhala K. , Belfon K. A. A. , Raguette L. , Huang H. , Migues A. N. , Bickel J. , Wang Y. , Pincay J. , Wu Q. , and Simmerling C. , ff19SB: Amino-Acid-Specific Protein Backbone Parameters Trained Against Quantum Mechanics Energy Surfaces in Solution, Journal of Chemical Theory and Computation. (2020) 16, no. 1, 528–552, 10.1021/acs.jctc.9b00591, 31714766. PMC13071887 31714766

[29] Roe D. R. and Cheatham T. E. , PTRAJ and CPPTRAJ: Software for Processing and Analysis of Molecular Dynamics Trajectory Data, Journal of Chemical Theory and Computation. (2013) 9, no. 7, 3084–3095, 10.1021/ct400341p, 26583988. 26583988

[30] Trott O. and Olson A. J. , AutoDock Vina: Improving the Speed and Accuracy of Docking With a New Scoring Function, Efficient Optimization, and Multithreading, Journal of Computational Chemistry. (2010) 31, no. 2, 455–461, 10.1002/jcc.21334, 19499576. PMC3041641 19499576

[31] Hucke F. I. L. and Bugert J. J. , Current and Promising Antivirals Against Chikungunya Virus, Frontiers in Public Health. (2020) 8, 618624, 10.3389/fpubh.2020.618624, 33384981. PMC7769948 33384981

[32] Guterres H. and Im W. , Improving Protein-Ligand Docking Results With High-Throughput Molecular Dynamics Simulations, Journal of Chemical Information and Modeling. (2020) 60, no. 4, 2189–2198, 10.1021/acs.jcim.0c00057, 32227880. PMC7534544 32227880

[33] Saha A. , Acharya B. N. , Priya R. , Tripathi N. K. , Shrivastava A. , Rao M. K. , Kesari P. , Narwal M. , Tomar S. , Bhagyawant S. S. , Parida M. , and Dash P. K. , Development of nsP2 Protease Based Cell Free High Throughput Screening Assay for Evaluation of Inhibitors Against Emerging Chikungunya Virus, Scientific Reports. (2018) 8, no. 1, 10831, 10.1038/s41598-018-29024-2, 30018455. PMC6050329 30018455

[34] Kalam N. , Ali R. , and Balasubramaniam V. R. , Exploring the Potential of Direct-Acting Antivirals Against Chikungunya Virus Through Structure-Based Drug Repositioning and Molecular Dynamic Simulations, Computers in Biology and Medicine. (2025) 189, 109989, 10.1016/j.compbiomed.2025.109989, 40056839. 40056839

[35] Narwal M. , Singh H. , Pratap S. , Malik A. , Kuhn R. J. , Kumar P. , and Tomar S. , Crystal Structure of Chikungunya Virus nsP2 Cysteine Protease Reveals a Putative Flexible Loop Blocking Its Active Site, International Journal of Biological Macromolecules. (2018) 116, 451–462, 10.1016/j.ijbiomac.2018.05.007, 29730006. 29730006

[36] Wu N. , Zhang R. , Peng X. , Fang L. , Chen K. , and Jestilä J. S. , Elucidation of Protein–Ligand Interactions by Multiple Trajectory Analysis Methods, Physical Chemistry Chemical Physics. (2024) 26, no. 8, 6903–6915, 10.1039/D3CP03492E, 38334015. 38334015

[37] Liu K. , Watanabe E. , and Kokubo H. , Exploring the Stability of Ligand Binding Modes to Proteins by Molecular Dynamics Simulations, Journal of Computer-Aided Molecular Design. (2017) 31, no. 2, 201–211, 10.1007/s10822-016-0005-2. 28074360

[38] Thomas S. , Rai J. , John L. , Günther S. , Drosten C. , Pützer B. M. , and Schaefer S. , Functional Dissection of the Alphavirus Capsid Protease: Sequence Requirements for Activity, Virology Journal. (2010) 7, no. 1, 10.1186/1743-422X-7-327, 21087473. PMC2999604 21087473

[39] Aggarwal M. , Sharma R. , Kumar P. , Parida M. , and Tomar S. , Kinetic Characterization of Trans-Proteolytic Activity of Chikungunya Virus Capsid Protease and Development of a FRET-Based HTS Assay, Scientific Reports. (2015) 5, no. 1, 14753, 10.1038/srep14753, 26439734. PMC4593962 26439734

[40] Raj A. , Sharmin S. , Ahmed Z. , Maha T. T. , Bishakha A. C. , Akter H. , Husna A. , Ripa F. A. , and Rumi F. , Harnessing Antiviral Peptides: From Molecular Mechanisms to Clinical Translation, Current Research in Pharmacology and Drug Discovery. (2025) 9, 100228, 10.1016/j.crphar.2025.100228, 40718095. PMC12296541 40718095

[41] de Castro S. and Camarasa M.-J. , Polypharmacology in HIV Inhibition: Can a Drug With Simultaneous Action Against Two Relevant Targets Be an Alternative to Combination Therapy?, European Journal of Medicinal Chemistry. (2018) 150, 206–227, 10.1016/j.ejmech.2018.03.007, 29529501. 29529501

[42] Pinzi L. , Tinivella A. , Caporuscio F. , and Rastelli G. , Drug Repurposing and Polypharmacology to Fight SARS-CoV-2 Through Inhibition of the Main Protease, Frontiers in Pharmacology. (2021) 12, 636989, 10.3389/fphar.2021.636989, 33692695. PMC7938350 33692695

[43] Tsiang M. , Jones G. S. , Goldsmith J. , Mulato A. , Hansen D. , Kan E. , Tsai L. , Bam R. A. , Stepan G. , Stray K. M. , Niedziela-Majka A. , Yant S. R. , Yu H. , Kukolj G. , Cihlar T. , Lazerwith S. E. , White K. L. , and Jin H. , Antiviral Activity of Bictegravir (GS-9883), a Novel Potent HIV-1 Integrase Strand Transfer Inhibitor with an Improved Resistance Profile, Antimicrobial Agents and Chemotherapy. (2016) 60, no. 12, 7086–7097, 10.1128/AAC.01474-16, 27645238. PMC5118987 27645238

