# Supplementary material for: Dual‐Target Repurposing Against Chikungunya Virus: Molecular Docking and Dynamics Reveal Bictegravir and Oxacillin as Promising Leads
Source: Biomed Res Int. 2026 Nov 4;2026:1672620. doi: 10.1155/bmri/1672620 (PMC13732648; doi:10.1155/bmri/1672620)
Supplement: Supplementary file 1 [file BMRI-2026-1672620-s001.docx]

**Table S1:** Docking scores of 202 FDA and internationally approved drugs against CHIKV nsp2 protease and capsid protein.

| **Compound (Sl_Name_CID)** | **Therapeutic Class** | **Regulatory / Approval Status** | **nsp2 docking score** | **Capsid docking score** |
| --- | --- | --- | --- | --- |
| E-64 (Reference ligand for nsp2) | - | - | -6.1 | - |
| Ethyl 3-aminobenzoate (Reference ligand for capsid) | - | - | - | -6.0 |
| 1_Abacavir_441300 | Antiviral | FDA Approved (USA) | -6.7 | -7 |
| 2_acetosulfone_31400 | Antibacterial | FDA Approved (USA) | -6.7 | -7.3 |
| 3_Acyclovir_135398513 | Antiviral | FDA Approved (USA) | -5.9 | -6.6 |
| 4_Adefovir_Dipivoxil_60871 | Antiviral | FDA Approved (USA) | -6.3 | -6.4 |
| 5_amantadine_2130 | Antiviral | FDA Approved (USA) | -4.7 | -5.8 |
| 6_amikacin_37768 | Antibacterial | FDA Approved (USA) | -6.3 | -5.9 |
| 7_Aminosalicylic_acid_4649 | Antibacterial | FDA Approved (USA) | -5.7 | -6 |
| 8_Amoxicillin_33613 | Antibacterial | FDA Approved (USA) | -6.9 | -6.9 |
| 9_Ampicillin_6249 | Antibacterial | FDA Approved (USA) | -6.1 | -6.5 |
| 10_anisomycin_253602 | Antibacterial | Investigational / Research Chemical | -6 | -6.3 |
| 11_Lauryldimethyl-3_4-dichlorobenzylammonium_7606 | Antibacterial | FDA Approved (USA) | -5.3 | -5 |
| 12_Atazanavir_148192 | Antiviral | FDA Approved (USA) | -7.2 | -6 |
| 13_Azithromycin_447043 | Antibacterial | FDA Approved (USA) | -7 | -5.1 |
| 14_Aztreonam_5742832 | Antibacterial | FDA Approved (USA) | -7 | -6 |
| 15_Baloxavir_Marboxil_124081896 | Antiviral | FDA Approved (USA) | -8 | -7.6 |
| 16_Bedaquiline_5388906 | Antibacterial | FDA Approved (USA) | -6.9 | -6.9 |
| 17_Benzoylpas_10718 | Antibacterial | FDA Approved (USA) | -7.2 | -7.4 |
| 18_Berberine_2353 | Antibacterial | OTC Supplement / EMA Monograph | -7.6 | -6.7 |
| 19_Besifloxacin_10178705 | Antibacterial | FDA Approved (USA) | -6.9 | -7.6 |
| 20_Bictegravir_90311989 | Antiviral | FDA Approved (USA) | -9.5 | -7.9 |
| 21_Biphenamine_21720 | Antifungal | FDA Approved (USA) | -6.6 | -6.5 |
| 22_Brincidofovir_483477 | Antiviral | FDA Approved (USA) | -4.8 | -6.3 |
| 23_carbomycin_6433412 | Antibacterial | Veterinary / Research Antibiotic | -7.4 | -5.9 |
| 24_Cefaclor_51039 | Antibacterial | FDA Approved (USA) | -6.9 | -6.9 |
| 25_Cefamandole_Nafate_23665731 | Antibacterial | FDA Approved (USA) | -6.9 | -7.5 |
| 26_Cefazolin_33255 | Antibacterial | FDA Approved (USA) | -6.7 | -6.4 |
| 27_Cefdinir_6915944 | Antibacterial | FDA Approved (USA) | -6.4 | -6.4 |
| 28_Cefepime_5479537 | Antibacterial | FDA Approved (USA) | -6.2 | -5.9 |
| 29_Cefiderocol_77843966 | Antibacterial | FDA Approved (USA) | -7.5 | -7.4 |
| 30_cefixime_5362065 | Antibacterial | FDA Approved (USA) | -6.6 | -5.9 |
| 31_Cefoperazone_44187 | Antibacterial | FDA Approved (USA) | -8 | -5.8 |
| 32_Cefotaxime_5742673 | Antibacterial | FDA Approved (USA) | -6.8 | -6 |
| 33_Cefotetan_53025 | Antibacterial | FDA Approved (USA) | -6.2 | -6.3 |
| 34_Cefoxitin_441199 | Antibacterial | FDA Approved (USA) | -5.7 | -6 |
| 35_Cefpodoxime_6335986 | Antibacterial | FDA Approved (USA) | -6.7 | -5.6 |
| 36_Cefprozil_5281006 | Antibacterial | FDA Approved (USA) | -6.7 | -6.3 |
| 37_Ceftaroline_9852981 | Antibacterial | FDA Approved (USA) | -7.9 | -6.7 |
| 38_Ceftazidime_5481173 | Antibacterial | FDA Approved (USA) | -7 | -6.8 |
| 39_Ceftolozane_49863420 | Antibacterial | FDA Approved (USA) | -7.9 | -6 |
| 40_Ceftriaxone_5479530 | Antibacterial | FDA Approved (USA) | -6.7 | -7.1 |
| 41_Cefuroxime_5479529 | Antibacterial | FDA Approved (USA) | -6.1 | -6.9 |
| 42_Cefacetrile_91562 | Antibacterial | EMA Approved (Veterinary) | -5.3 | -5.6 |
| 43_Chloramine T_3641960 | Antibacterial | FDA Approved (USA) | -6.2 | -6.2 |
| 44_Chloramphenicol_palmitate_443382 | Antibacterial | FDA Approved (USA) | -5 | -5.4 |
| 45_Chloramphenicol_succinate_656580 | Antibacterial | FDA Approved (USA) | -6.2 | -7.1 |
| 46_Chloroxylenol_2723 | Antibacterial | FDA Approved (USA) | -4.8 | -5.6 |
| 47_Chlorquinaldol_6301 | Antibacterial | EMA / Swissmedic Approved | -6 | -6.8 |
| 48_cidofovir_60613 | Antiviral | FDA Approved (USA) | -5.9 | -6.4 |
| 49_Cilastatin_6435415 | Antibacterial | FDA Approved (USA) | -5.8 | -6.6 |
| 50_Cimicifugin_45048779 | Anti-inflammatory | Natural Bioactive / Research Chemical | -8.4 | -7.7 |
| 51_Clarithromycin_84029 | Antibacterial | FDA Approved (USA) | -6.5 | -5.7 |
| 52_Clavulanic_Acid_5280980 | Antibacterial | FDA Approved (USA) | -5.5 | -6.9 |
| 53_clindamycin_446598 | Antibacterial | FDA Approved (USA) | -5.6 | -5.8 |
| 54_Cobicistat_25151504 | Antiviral | FDA Approved (USA) | -7.1 | -5.9 |
| 55_m-cresol_342 | Antibacterial | FDA Approved (USA) | -4.7 | -5.1 |
| 56_Cycloserine_6234 | Antibacterial | FDA Approved (USA) | -3.9 | -4.2 |
| 57_Dalfopristin_6323289 | Antibacterial | FDA Approved (USA) | -7.2 | -6.4 |
| 58_Dapsone_2955 | Antibacterial | FDA Approved (USA) | -6.2 | -7.3 |
| 59_Darunavir_213039 | Antiviral | FDA Approved (USA) | -7 | -6.4 |
| 60_Delafloxacin_487101 | Antibacterial | FDA Approved (USA) | -7.4 | -6.5 |
| 61_Demeclocycline_54680690 | Antibacterial | FDA Approved (USA) | -7 | -7.5 |
| 62_Dequalinium_2993 | Antibacterial | EMA / MHRA / Health Canada Approved | -6.9 | -6.4 |
| 63_Dichlorophene_3037 | Antiparasitic | FDA Approved (USA) | -6.1 | -7.3 |
| 64_Dicloxacillin_18381 | Antibacterial | FDA Approved (USA) | -6.7 | -6.4 |
| 65_Docosanol_12620 | Antiviral | FDA Approved (USA) | -5.3 | -4.8 |
| 66_Dolutegravir_54726191 | Antiviral | FDA Approved (USA) | -8 | -7.5 |
| 67_Domiphen_3149 | Antibacterial | FDA Approved (USA) | -4.9 | -5.2 |
| 68_Doravirine_58460047 | Antiviral | FDA Approved (USA) | -8.1 | -6.9 |
| 69_Doripenem_73303 | Antibacterial | FDA Approved (USA) | -6.1 | -6.7 |
| 70_Doxorubicin_31703 | Antineoplastic | FDA Approved (USA) | -8 | -6.4 |
| 71_Doxycycline_54671203 | Antibacterial | FDA Approved (USA) | -6.5 | -7.1 |
| 72_Efavirenz_64139 | Antiviral | FDA Approved (USA) | -6.1 | -6.1 |
| 73_Elbasvir_11574 | Antiviral | FDA Approved (USA) | -8.4 | -7.3 |
| 74_Elvitegravir_5277135 | Antiviral | FDA Approved (USA) | -7.7 | -8.3 |
| 75_Entecavir_135398508 | Antiviral | FDA Approved (USA) | -6.2 | -6.6 |
| 76_Eravacycline_54726192 | Antibacterial | FDA Approved (USA) | -8.5 | -6.7 |
| 77_Ertapenem_150610 | Antibacterial | FDA Approved (USA) | -7.3 | -7.4 |
| 78_Erythromycin_12560 | Antibacterial | FDA Approved (USA) | -6.7 | -5.5 |
| 79_Ethaverine_3280 | Smooth Muscle Relaxant | FDA Approved (USA) | -6.6 | -6.2 |
| 80_Ethionamide_2761171 | Antibacterial | FDA Approved (USA) | -5.4 | -5.2 |
| 81_Etravirine_193962 | Antiviral | FDA Approved (USA) | -7 | -6.8 |
| 82_Famciclovir_3324 | Antiviral | FDA Approved (USA) | -6.3 | -5.9 |
| 83_Fidaxomicin_70678896 | Antibacterial | FDA Approved (USA) | -8.4 | -4.7 |
| 84_Finafloxacin_11567473 | Antibacterial | FDA Approved (USA) | -7 | -7.8 |
| 85_Floxuridine_5790 | Antineoplastic | FDA Approved (USA) | -5.8 | -6.9 |
| 86_Formic acid_284 | Antibacterial | FDA Approved (USA) | -3.1 | -2.6 |
| 87_Fosamprenavir_131536 | Antiviral | FDA Approved (USA) | -6.7 | -6.3 |
| 88_Foscarnet_3415 | Antiviral | FDA Approved (USA) | -4.3 | -4.6 |
| 89_Fosfomycin_446987 | Antibacterial | FDA Approved (USA) | -4.2 | -4.7 |
| 90_Fostemsavir_11319217 | Antiviral | FDA Approved (USA) | -7.6 | -7.4 |
| 91_Ganciclovir_135398740 | Antiviral | FDA Approved (USA) | -5.4 | -6.7 |
| 92_Gatifloxacin_5379 | Antibacterial | FDA Approved (USA) | -6.4 | -6.1 |
| 93_Gemifloxacin_9571107 | Antibacterial | FDA Approved (USA) | -7 | -7.3 |
| 94_Gentamicin_3467 | Antibacterial | FDA Approved (USA) | -6.9 | -6.1 |
| 95_Glecaprevir_66828839 | Antiviral | FDA Approved (USA) | -9.2 | -8.5 |
| 96_Glucosulfone_487090 | Antibacterial | FDA Approved (USA) | -7.8 | -7.2 |
| 97_Grazoprevir_44603531 | Antiviral | FDA Approved (USA) | -9.1 | -6.8 |
| 98_Haloprogin_3561 | Antifungal | FDA Approved (USA) | -4.7 | -5.3 |
| 99_Imipenem_104838 | Antibacterial | FDA Approved (USA) | -5.8 | -6.8 |
| 100_Imiquimod_57469 | Immunomodulator | FDA Approved (USA) | -6.5 | -7 |
| 101_Indinavir_5362440 | Antiviral | FDA Approved (USA) | -8.5 | -7.6 |
| 102_Iodoform_6374 | Antibacterial | FDA Approved (USA) | -2.5 | -2.5 |
| 103_Isocyclamine_7743 | Vasodilator | Investigational / Research Chemical | -4.6 | -5.1 |
| 104_Isoniazid_3767 | Antibacterial | FDA Approved (USA) | -5.3 | -5.9 |
| 105_Kanamycin_6032 | Antibacterial | FDA Approved (USA) | -6.2 | -6.1 |
| 106_Lamivudine_60825 | Antiviral | FDA Approved (USA) | -5.6 | -5.9 |
| 107_Ledipasvir_67505836 | Antiviral | FDA Approved (USA) | -9 | -7.8 |
| 108_Lefamulin_25185057 | Antibacterial | FDA Approved (USA) | -6.6 | -6.5 |
| 109_Letermovir_45138674 | Antiviral | FDA Approved (USA) | -7.1 | -6.7 |
| 110_Levofloxacin_149096 | Antibacterial | FDA Approved (USA) | -7.1 | -7.9 |
| 111_Lincomycin_3000540 | Antibacterial | FDA Approved (USA) | -5.8 | -6.2 |
| 112_Linezolid_441401 | Antibacterial | FDA Approved (USA) | -6.6 | -7.3 |
| 113_Lopinavir_92727 | Antiviral | FDA Approved (USA) | -7.6 | -6.7 |
| 114_Mafenide_3998 | Antibacterial | FDA Approved (USA) | -5.5 | -5.9 |
| 115_Maraviroc_3002977 | Antiviral | FDA Approved (USA) | -8.3 | -7.9 |
| 116_Maribavir_471161 | Antiviral | FDA Approved (USA) | -6.5 | -6.1 |
| 117_Meropenem_441130 | Antibacterial | FDA Approved (USA) | -6.5 | -6.1 |
| 118_Methacycline_54675785 | Antibacterial | FDA Approved (USA) | -7.2 | -7 |
| 119_Methenamine_4101 | Antibacterial | FDA Approved (USA) | -3.3 | -4.7 |
| 120_Mezlocillin_656511 | Antibacterial | FDA Approved (USA) | -8.3 | -7.1 |
| 121_Minocycline_54675783 | Antibacterial | FDA Approved (USA) | -7.5 | -7.2 |
| 122_Moxifloxacin_152946 | Antibacterial | FDA Approved (USA) | -6.9 | -6.5 |
| 123_Mupirocin_446596 | Antibacterial | FDA Approved (USA) | -5.8 | -5.8 |
| 124_Lapyrium_22660 | Antibacterial | FDA Approved (USA) | -6.7 | -5.9 |
| 125_Nafcillin_8982 | Antibacterial | FDA Approved (USA) | -7.1 | -7.1 |
| 126_Nelfinavir_64143 | Antiviral | FDA Approved (USA) | -7.4 | -6.4 |
| 127_Nevirapine_4463 | Antiviral | FDA Approved (USA) | -6.5 | -6.5 |
| 128_Nifuroxime_6478035 | Antibacterial | FDA Approved (USA) | -5.6 | -6.4 |
| 129_Nifurtimox_6842999 | Antiparasitic | FDA Approved (USA) | -6 | -7 |
| 130_Nitrofurantoin_6604200 | Antibacterial | FDA Approved (USA) | -6.1 | -7 |
| 131_*p*-Nitrosulfathiazole_10127 | Antibacterial | FDA Approved (USA) | -6 | -7.4 |
| 132_Norfloxacin_4539 | Antibacterial | FDA Approved (USA) | -7.4 | -8 |
| 133_Novobiocin_54675769 | Antibacterial | FDA Approved (USA) | -8.4 | -7.4 |
| 134_Ofloxacin_4583 | Antibacterial | FDA Approved (USA) | -7.7 | -7.5 |
| 135_Omadacycline_54697325 | Antibacterial | FDA Approved (USA) | -6.9 | -6.7 |
| 136_Oseltamivir_65028 | Antiviral | FDA Approved (USA) | -5.4 | -6.3 |
| 137_Oxacillin_6196 | Antibacterial | FDA Approved (USA) | -6.7 | -8.7 |
| 138_Oxolinic_Acid_4628 | Antibacterial | EMA / PMDA Approved (Veterinary) | -7 | -8.1 |
| 139_Ozenoxacin_9863827 | Antibacterial | FDA Approved (USA) | -7.2 | -7.9 |
| 140_Paritaprevir_45110509 | Antiviral | FDA Approved (USA) | -8.8 | -8.6 |
| 141_Paromomycin_165580 | Antiparasitic | FDA Approved (USA) | -6.1 | -6.8 |
| 142_Penciclover_135398748 | Antiviral | FDA Approved (USA) | -6.1 | -6.5 |
| 143_Penicillin G_5904 | Antibacterial | FDA Approved (USA) | -6.3 | -6.7 |
| 144_Penicillin V_6869 | Antibacterial | FDA Approved (USA) | -6.8 | -6.9 |
| 145_Pentylphenol_61084 | Antibacterial | Investigational / Disinfectant | -5.7 | -5.7 |
| 146_Peramivir_154234 | Antiviral | FDA Approved (USA) | -5.6 | -5.7 |
| 147_Pheneticillin_272833 | Antibacterial | MHRA / EMA Approved | -6.8 | -6.8 |
| 148_Phthalylsulfathiazole_4806 | Antibacterial | EMA / Pharmacopeial Approved | -7.3 | -7.9 |
| 149_Piperacillin_43672 | Antibacterial | FDA Approved (USA) | -7.8 | -7.3 |
| 150_Plazomicin_42613186 | Antibacterial | FDA Approved (USA) | -6.6 | -5.5 |
| 151_Podofilox_10607 | Antiviral | FDA Approved (USA) | -6.7 | -6.6 |
| 152_Pretomanid_456199 | Antibacterial | FDA Approved (USA) | -7 | -7.2 |
| 153_Pyrazinamide_1046 | Antibacterial | FDA Approved (USA) | -4.9 | -4.7 |
| 154_Quinupristin_5388937 | Antibacterial | FDA Approved (USA) | -8.8 | -4.9 |
| 155_Raltegravir_54671008 | Antiviral | FDA Approved (USA) | -8.5 | -7.4 |
| 156_Remdesivir_121304016 | Antiviral | FDA Approved (USA) | -7.4 | -7.2 |
| 157_Retapamulin_6918462 | Antibacterial | FDA Approved (USA) | -6.6 | -6.8 |
| 158_Rifabutin_6323490 | Antibacterial | FDA Approved (USA) | -7.9 | -7 |
| 159_Rifampin_135398735 | Antibacterial | FDA Approved (USA) | -7.1 | -4.4 |
| 160_Rifamycin_6324616 | Antibacterial | FDA Approved (USA) | -7.4 | -6.2 |
| 161_Rifapentine_135403821 | Antibacterial | FDA Approved (USA) | -9.1 | -7.5 |
| 162_Rifaximin_6436173 | Antibacterial | FDA Approved (USA) | -8 | -5.2 |
| 163_Rilpivirine_6451164 | Antiviral | FDA Approved (USA) | -7.5 | -7.9 |
| 164_Rimantadine_5071 | Antiviral | FDA Approved (USA) | -5.1 | -6.5 |
| 165_Ritonavir_392622 | Antiviral | FDA Approved (USA) | -7.8 | -6 |
| 166_Rolitetracycline_54682938 | Antibacterial | FDA Approved (USA) | -7.3 | -7.2 |
| 167_Rotenone_6758 | Antiparasitic | US EPA Approved (Pesticide) | -7.6 | -7.1 |
| 168_Saquinavir_441243 | Antiviral | FDA Approved (USA) | -9.1 | -7.4 |
| 169_Sarecycline_54681908 | Antibacterial | FDA Approved (USA) | -7 | -6.6 |
| 170_Simeprevir_24873435 | Antiviral | FDA Approved (USA) | -9.3 | -7.8 |
| 171_Sisomicin_36119 | Antibacterial | EMA / International Approved | -6.4 | -5.7 |
| 172_Sofosbuvir_45375808 | Antiviral | FDA Approved (USA) | -7.2 | -6.5 |
| 173_Streptomycin_19649 | Antibacterial | FDA Approved (USA) | -7 | -6.1 |
| 174_Succinylsulfathiazole_5315 | Antibacterial | EMA / Pharmacopeial Approved | -6.9 | -7.4 |
| 175_Sulfacetamide_5320 | Antibacterial | FDA Approved (USA) | -6.2 | -6.6 |
| 176_Sulfadiazine_5215 | Antibacterial | FDA Approved (USA) | -6.2 | -7.1 |
| 177_Sulfaethidole_7181 | Antibacterial | Veterinary / Pharmacopeial Approved | -6.1 | -7.1 |
| 178_Sulfaguanidine_5324 | Antibacterial | EMA / Pharmacopeial Approved | -6 | -6.5 |
| 179_Sulfalene_9047 | Antibacterial | EMA / WHO Approved | -6.3 | -7.3 |
| 180_Sulfamerazine_5325 | Antibacterial | FDA Approved (USA) | -6.5 | -6.6 |
| 181_Sulfanilamide_5333 | Antibacterial | FDA Approved (USA) | -5.3 | -5.5 |
| 182_Sulfapyrazine_8309 | Antibacterial | PMDA / MAFF Approved (Veterinary) | -6.2 | -7.4 |
| 183_Sulfisomidine_5343 | Antibacterial | EMA / Swissmedic Approved | -6.2 | -7.5 |
| 184_Tazobactam_123630 | Antibacterial | FDA Approved (USA) | -5.3 | -7 |
| 185_Tecovirimat_16124688 | Antiviral | FDA Approved (USA) | -8.4 | -8 |
| 186_Tedizolid_Phosphate_11476460 | Antibacterial | FDA Approved (USA) | -7.7 | -7.7 |
| 187_Tenofovir_alafenamide_9574768 | Antiviral | FDA Approved (USA) | -6.3 | -6.8 |
| 188_Tenofovir_Disoproxil_5481350 | Antiviral | FDA Approved (USA) | -6.3 | -5.8 |
| 189_Tetracycline_54675776 | Antibacterial | FDA Approved (USA) | -6.6 | -7.3 |
| 190_Thiazosulfone_10124 | Antibacterial | Investigational / Historical Antimicrobial | -5.9 | -7.1 |
| 191_Thymol_6989 | Antibacterial | FDA Approved (USA) | -5.9 | -5.9 |
| 192_Tigecycline_54686904 | Antibacterial | FDA Approved (USA) | -7.6 | -6.6 |
| 193_Tipranavir_54682461 | Antiviral | FDA Approved (USA) | -6.3 | -7.2 |
| 194_Tobramycin_36294 | Antibacterial | FDA Approved (USA) | -5.6 | -6.1 |
| 195_Trifluridine_6256 | Antiviral | FDA Approved (USA) | -6.3 | -7.4 |
| 196_Trimethoprim_5578 | Antibacterial | FDA Approved (USA) | -6 | -7.1 |
| 197_Trovafloxacin_62959 | Antibacterial | FDA Approved (USA) | -8 | -8.8 |
| 198_Valganciclovir_135413535 | Antiviral | FDA Approved (USA) | -6.3 | -6.6 |
| 199_Velpatasvir_67683363 | Antiviral | FDA Approved (USA) | -8.8 | -7.4 |
| 200_voxilaprevir_89921642 | Antiviral | FDA Approved (USA) | -7.9 | -8.5 |
| 201_Zanamivir_60855 | Antiviral | FDA Approved (USA) | -5.8 | -6.9 |
| 202_Zidovudine_35370 | Antiviral | FDA Approved (USA) | -6.5 | -7.5 |

CID, PubChem Compound Identifier; EMA, European Medicines Agency (European Union); FDA, Food and Drug Administration (United States); MAFF, Ministry of Agriculture, Forestry and Fisheries (Japan); MHRA, Medicines and Healthcare products Regulatory Agency (United Kingdom); OTC, Over-the-Counter; PMDA, Pharmaceuticals and Medical Devices Agency (Japan); Swissmedic, Swiss Agency for Therapeutic Products (Switzerland); US EPA, United States Environmental Protection Agency; WHO, World Health Organization.
